# Supplementary material for: HIV-1 single-transcription start-site mutants display complementary replication functions that are restored by reversion
Source: J Virol. 2025 Mar 4;99(4):e02139-24. doi: 10.1128/jvi.02139-24 (PMC11998490; doi:10.1128/jvi.02139-24)
Supplement: Supplemental material — Table S1; Figures S1 and S2. [file jvi.02139-24-s0001.pdf]

Supplementary Table 1

Descriptive Statistics (MS2-mCherry)

Sample	Mean	Median	Std. Error of Mean	Lower 95% CI of Mean	Upper 95% CI of Mean
ΔNC	0.05	0.19	0.06	0.00	0.16
WT	70.45	60.27	0.16	70.14	70.75
^{cap}1G	74.32	62.95	0.15	74.03	74.62
^{cap}3G	54.03	43.22	0.14	53.77	54.30

Descriptive Statistics (Gag-YFP)

Sample	Mean	Median	Std. Error of Mean	Lower 95% CI of Mean	Upper 95% CI of Mean
ΔNC	606.5	565.5	1.80	602.9	610
WT	1212	1124	1.23	1210	1214
^{cap}1G	1105	1012	1.00	1104	1107
^{cap}3G	1440	1354	1.30	1437	1442

Descriptive Statistics (MS2-mCherry/Gag-YFP)

Sample	Mean	Median	Std. Error of Mean	Lower 95% CI of Mean	Upper 95% CI of Mean
ΔNC	0.000	0.000	0.00	0.000	0.000
WT	0.059	0.053	0.00	0.058	0.059
^{cap}1G	0.065	0.058	0.00	0.065	0.066
^{cap}3G	0.038	0.033	0.00	0.037	0.038

Table S1. Corresponding statistical values to SVA data

Values for mean, median, standard error of the mean, lower 95% confidence interval, and upper 95% confidence interval are shown for ΔNC, WT, ^{cap}1G, and ^{cap}3G virions for corresponding MS2-mCherry, Gag-YFP, and MS2-mCherry/Gag-YFP fluorescence, respectively.

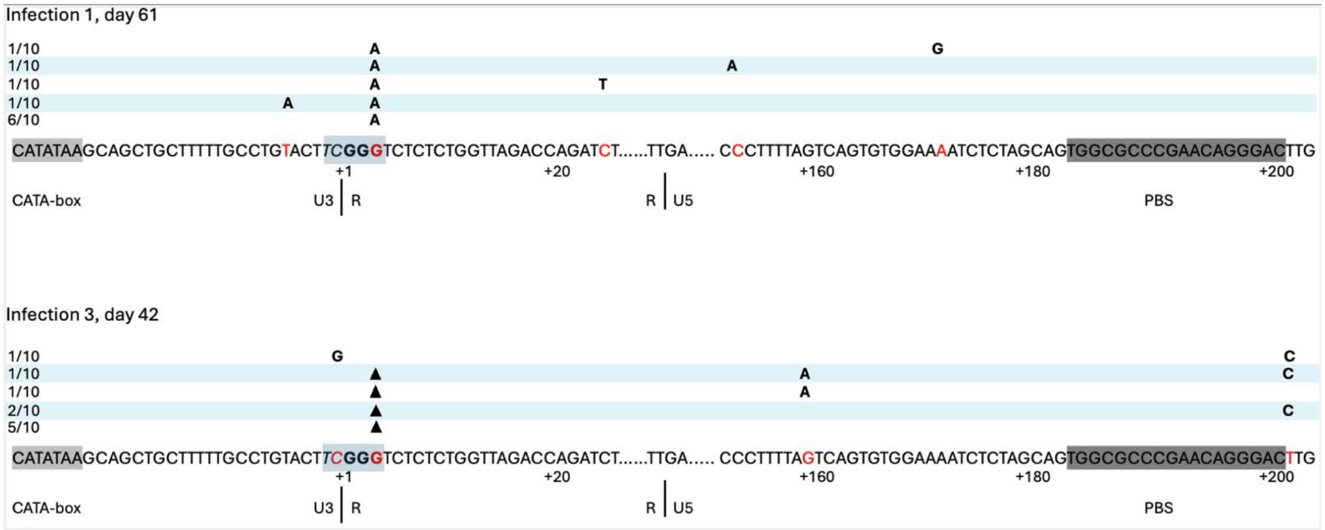

Fig. S1. Mutations observed in the proviral DNA subclones obtained from late timepoint of MT4 cell infections with 3G-only virus. Data shown is from 10 sequenced subclones for each revertant, with the prevalence of each revertant sequence indicated by N/10. Nucleotides in the ancestor sequence that changed are shown in red. GGG nucleotides at the TSS are shown in bold and TC dinucleotide insertion responsible for 3G-only phenotype is in italic. Observed mutations are shown in bold above mutated nucleotides. Black triangles indicate observed nucleotide deletion.

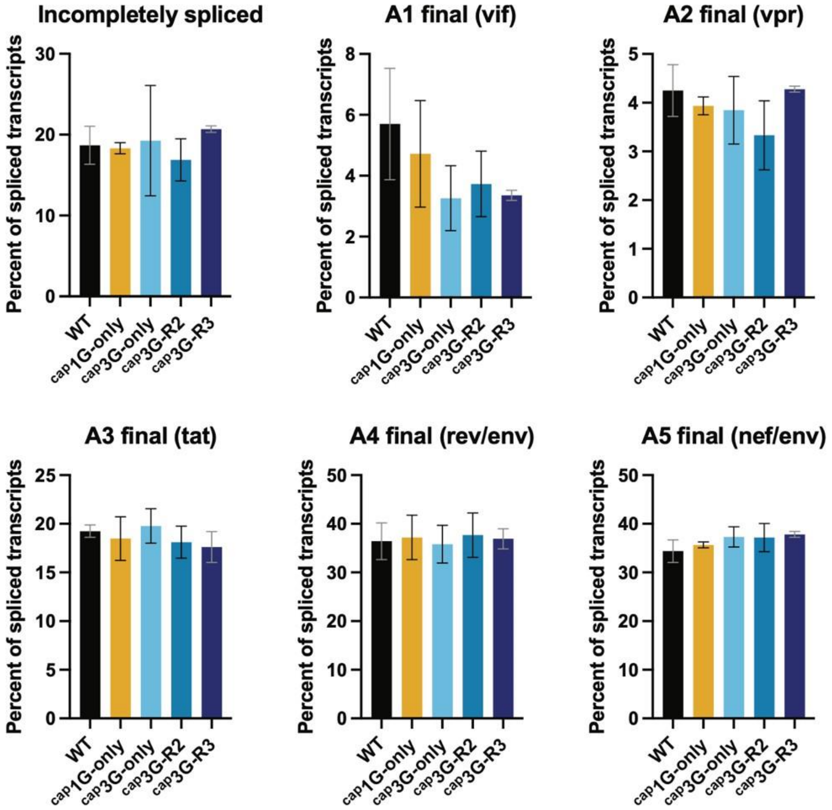

Fig. S2. Percent of the individual spliced viral RNA species in the MT4 cells infected with HIV-1 TSS variants as assessed by high throughput sequencing analysis. Designations are as indicated in the legend to Fig. 8.
